# “Show me how to use a microscope” – The development and evaluation of certification as direct assessment of practical lab skills

**DOI:** 10.1002/ece3.10592

**Published:** 2023-10-11

**Authors:** Pernille Bronken Eidesen, Anne E. Bjune, Simone I. Lang

**Affiliations:** ^1^ Department of Biosciences University of Oslo Oslo Norway; ^2^ Department of Arctic Biology The University Centre in Svalbard Longyearbyen Norway; ^3^ Department of Biological Sciences (BIO) University of Bergen Bergen Norway

**Keywords:** constructive alignment, direct assessment, learning, microscopy, peer‐assessment, practical skills

## Abstract

Practical lab skills are rarely directly assessed. To improve constructive alignment between the described learning outcomes of practical skills and assessment, we developed and tested a certification procedure for microscopy skills. The procedure was embedded into the ordinary learning activity, so no additional time was needed. Three slightly different protocols were developed within the framework of sociocultural learning theory and built like a skill ladder, including direct peer assessment and elements of gamified learning. The protocols varied slightly in the way students were prepared for the certification, the number of steps/levels of achievement, and the consequences of failing. We tested the protocols at three different academic institutions and within 11 courses of varying sizes and academic levels in biology or geology. Feedbacks were collected through online surveys (*n* = 207) or orally after sessions. One protocol provided instruction videos as preparation material. Instruction videos provided increased understanding of the task, but tactile training was most important for learning. Regardless of institution, type of preparation, and level of former experience, the certification procedure made students clearly more engaged in the exercise. The majority reported that the certification procedure increased their motivation to learn, increased their perceived learning outcome, and was appropriate for assessing practical skills. Students with no or little experience in microscopy before the exercise were more positive about the certification procedure compared to skilled students, and the level of engagement and preparation was higher when there were some consequences of failing. Most students felt comfortable being certified by peers, but some students expressed concern about peers making mistakes. The presented certification procedure can easily be adapted to assess other practical skills and, with some adjustments, be an efficient method for assessment‐as‐learning, merging formative‐ and summative assessment.

## Graphical abstract

Practical lab skills are rarely directly assessed. We developed and tested a certification procedure for microscopy skills resulting in higher motivation to learn, and perceived better learning outcome.

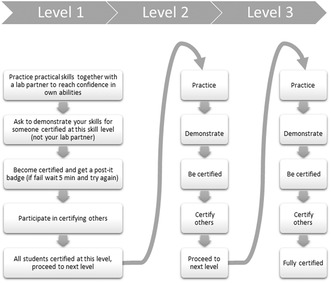

## 1 BACKGROUND

During the last decade, John Biggs' principle of constructive alignment has emerged as the gold standard for course design in higher education (Biggs, 1996; Biggs & Tang, 2011), and research supports that good constructive alignment enhances learning (Adams, 2020; Biggs et al., 2022; Brabrand, 2008). To achieve constructive alignment, you should start formulating your intended learning outcomes (ILOs) and subsequently align your teaching content and assessment to those ILOs (Biggs & Tang, 2011). However, ILOs related to practical skills are often misaligned (Adams, 2020). For instance, to achieve the ILO to ‘use a microscope’, the learning activity usually includes ‘using’ a microscope, but the assessment is not aligned with the ‘use’ itself. It is aligned to the result of the use and assessed indirectly through lab reports of the type ‘draw and describe what you see’ and link the observations to theory. These types of indirect assessments do address learning outcomes related to knowledge content but do not assess practical lab skills (Abrahams & Reiss, 2015). To improve the constructive alignment of ILOs of practical lab skills, we need to rethink how we plan and perform our lab activities and, in particular, the associated assignments.

A shift from indirect to direct assessment is, however, not sufficient to improve the constructive alignment of practical lab skills. Constructive alignment requires attention to what students do. Biggs outlined constructive alignment within the pedagogical framework of constructivism. Although “Constructivism refers to a rather loose and fuzzy collection of approaches [..]”, the core idea is that learners in some way actively construct knowledge (van Geert, 2017). Biggs emphasizes that it is what students *do* that is important for learning – the students themselves must be actively engaged in learning experiences rather than passively receiving information (Biggs, 1996; Loughlin et al., 2021). Along with the shift towards more student‐active pedagogies and a focus on skills and competences, the division into formative and summative assessment has also been challenged, and blended solutions have emerged (Bailey et al., 2017; Broadbent et al., 2018). We are moving from assessment‐of‐learning (summative), through assessment‐for‐learning (formative), to a blended form referred to as assessment‐as‐learning (Earl, 2012; Yan & Boud, 2022).

Interaction with peers can be an efficient way to actively engage students in learning activities (Crouch & Mazur, 2001). Social constructivism is a branch of constructivism emphasizing that learning is dependent on interactions with others. This is in line with Lev Vygotsky's sociocultural learning theory, which underscores the importance of social interaction in the cognitive learning work and the expansion of the individual's zone of proximal development (Imsen, 2016, pp. 192–193; Vygotsky et al., 1978). The latter refers to the distance between what a learner can manage alone (actual performance level) and what a learner can do under active guidance and interaction with more advanced peers (potential performance level). In other words, you learn more, and your learning‐potential increases through social interaction. The same effect is seen when applied in assessment settings. In courses where students are engaged in peer‐review processes as part of the assessment, the students experience, for example, deeper learning of discipline knowledge (Harland et al., 2017; Reddy et al., 2021).

The impact of assessment per se is also directional for learning; students are more likely to put effort into learning outcomes that are assessed (Biggs & Tang, 2011; Brown & Hirschfeld, 2008; Hattie, 2015), and the possibility of getting feedback motivates preparation and learning (Burgess et al., 2020; Shepard, 2000). Thus, if practical skills are important to learn, direct assessments should be prioritized (Abrahams & Reiss, 2015). Good ways of doing direct assessment exist but they are often only feasible for a small number of students. For instance, procedures for practical exams in chemistry have been successfully developed and tested but became challenging when the number of students exceeded 100 (Hancock & Hollamby, 2020; Kirton et al., 2014). Direct assessments of practical lab skills are also common within clinical educations like medicine or biomedical laboratory science (Majumder et al., 2019; Wong & Devaiah, 2013), but the student numbers are usually low and more resources are available per student. The challenge lies in performing well‐aligned, direct assessments of practical lab skills in a cost and time‐efficient manner.

A promising approach tested in chemistry was using performance videos in combination with digital badges (Hennah & Seery, 2017; Towns et al., 2015). The possibility of achieving a digital badge created an element of positive competition in the class; they wanted to perform well (Hennah & Seery, 2017). Introducing common gaming elements in educational settings, like badges and achievement levels (i.e., gamification; e.g., Bai et al., 2020; Rutledge et al., 2018), fosters enthusiasm and is appreciated by students due to the possibility of providing feedback on performance (Bai et al., 2020).

We took up the challenge and designed and tested a certification procedure for the basic use of a light microscope. The backbone of the certification procedure was inspired by typical certification procedures commonly used in vocational education (such as the practical assessment for a plumber (OECD, 2022 Annex 5.B.)). Further, we aimed at including elements that enhance learning in the certification procedure, that is, elements that motivate, activate, create variation, provide feedback, create individual responsibility, interaction, and cooperation with peers (Biggs & Tang, 2011; Hiim & Hippe, 2009; Vygotsky et al., 1978). The implementation of peer instruction and peer assessment techniques would expectantly also lead to greater time efficiency. We present how the certification procedure was developed in relation to pedagogical theory, how it was used, and examples of protocols. Based on the collected feedback, we evaluate and discuss our results, considering relevant research and pedagogical theory. We address (1) whether the developed certification procedure increases alignment and perceived learning; (2) possible pitfalls and ways to mitigate them; and (3) if efficient, how the certification procedure can be adjusted and applied to assess other practical skills.

## 2 METHOD

### 2.1 Development of the general certification procedure

Initially, we made a well‐defined list of practical tasks aligned with our ILOs for light microscopy and distributed these tasks as a skill ladder with advancing difficulty. The workload was adjusted so students should be able to finish all steps (both practice time and certification of all steps) within a 2‐h practical.

The students were introduced to light microscopy and all tasks before the practical started. They were also provided with the full list of tasks they were expected to show during the certification (example given in Table 1). This list was used as the basis for the training activity, creating full alignment between practice and assessment (Biggs & Tang, 2011).

**TABLE 1:** Overview of test environments at University Centre in Svalbard (UNIS), University of Bergen (UiB), and University of Oslo (UiO). Academic level, time frame, number of courses and enrolled students, type of lab‐preparation, and certification protocol (number of levels tested during the lab) among the three institutions.

	UNIS	UiB	UiO	UiO
Academic level	From 2nd year bachelor to master* in biology or geology	3rd year bachelor biology	First‐year bachelor in biology	First‐year bachelor in biology
When	March 2021 to June 2022	2021 and 2022	2021	2022
Tested in	Eight different courses	Same course twice	Same course twice	
Number of students enrolled	10–20 per course, about 140 total	15 per year	114, divided in six groups	164, divided in six groups
Type of introduction/ preparation	Lecture as part of the 2 h lab session with certification	Lecture as part of the 2 h lab session with certification	Lecture (45 min) week before 2 h lab session with certification	Prerecorded instruction videos and a mandatory quiz with at least 10 of 12 points to be allowed into the lab
Protocol	Protocol with three levels in 2 h	Protocol with three levels in 2 h	Protocol with four levels in 2 h. Certifier had to sign the protocol	Protocol with three levels in 2 h. Certifier had to sign the protocol

* One course included five staff members.

Starting at skill‐level one, the students practiced in pairs on the practical tasks to be certified at level one and took turns to orally explain to each other what they were doing when performing the practical tasks and why. To reduce stress and account for individual differences, the students were given the freedom to practice with their partner until they felt confident and ready to be assessed [within a maximum time limit of 20 min]. When ready, they raised their hands and asked to demonstrate their skills for someone certified at this skill level. The first students were always certified by a teacher, but as the more skilled students were assumed to pass the certifications first, they were given the task of certifying others. In this way, the fastest students were kept active and expected to represent a mediating agent, expanding their peers' knowledge (Vygotsky et al., 1978). In addition, the use of peer assessment was expected to increase efficiency (Harris, 2011). To avoid loyalty issues (lab partners were often friends), we made a rule that lab partners were not allowed to certify each other. This was also expected to increase the individual responsibility and thus enhance learning (Cantillon & Macdermott, 2008).

If students failed at first attempt, they got a new chance, but they could not continue immediately with another round of certification. The certifier should move on, let the student practice once more, and then try again after 5 min. This was done to avoid students asking for certification before they were ready, just hoping to be lucky.

Inspired by gamification, the students were rewarded ‘badges’ in the form of a post‐it note of a certain colour when being certified at a given level. The post‐it note was added as a medal to the lab coat. This ‘badge’ showed that you were allowed to certify others at a given skill level and was expected to be motivating for some students (Bai et al., 2020; Rutledge et al., 2018).

When most students were certified at level one, they were allowed to start practising for the next level, and the procedure was repeated. If some students were still struggling, they got support from a teacher.

**FIGURE 1:**
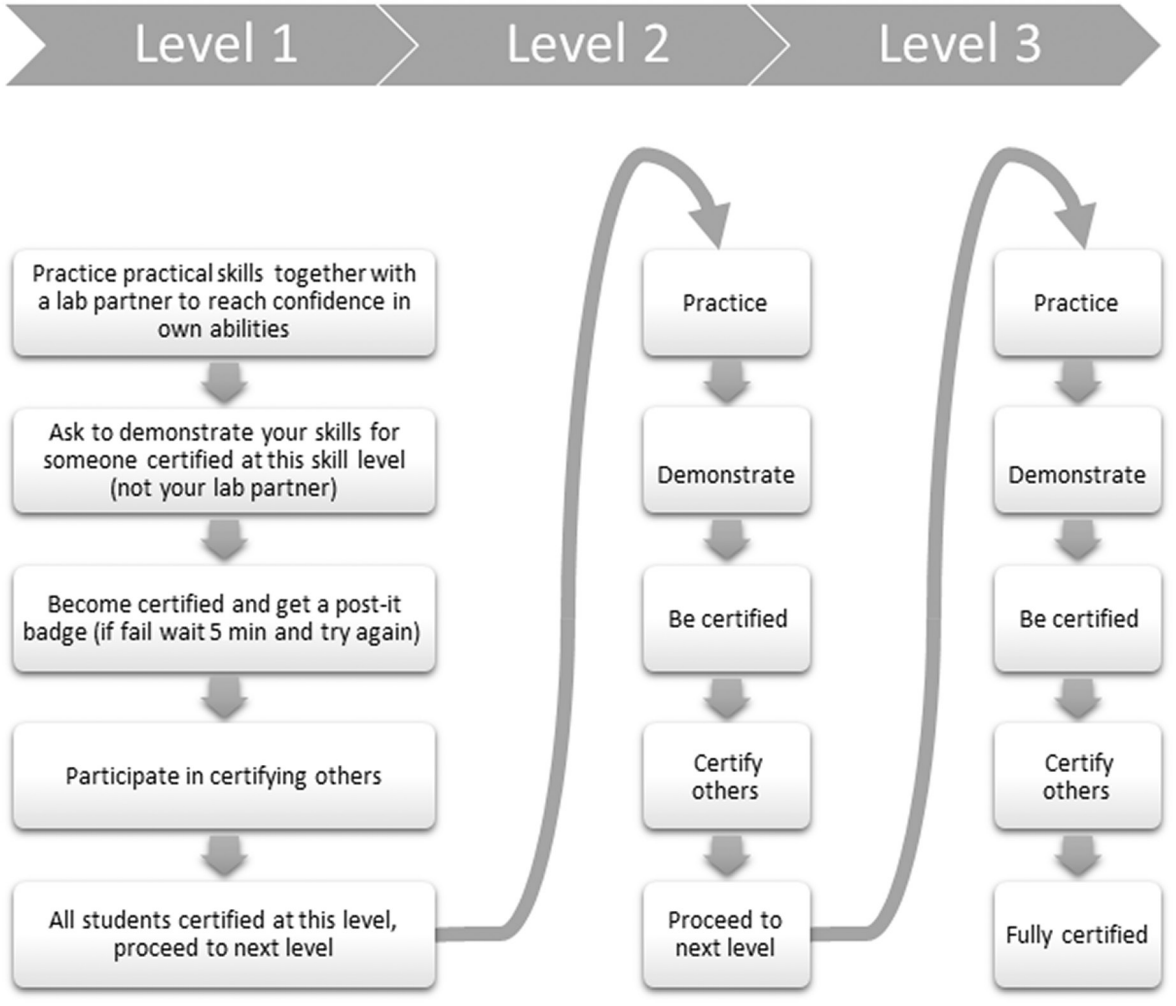
The certification procedure in microscopy followed a skill ladder, where the same procedure was repeated but with new tasks at a higher skill level. Each level consisted of: practice practical skills together with a lab partner to reach confidence in their own abilities; demonstrating skills for someone certified at this skill level (someone else than the lab partner); when certified, participating in certifying others. We used post‐it notes of different colours to indicate passing a skill level. After passing all required levels, students achieved a certificate stating their skill level.

Thus, the certification procedure and the practice for the certification were combined by alternating between practice time and assessment by certification at each step of the procedure. The lab exercise was approved when the student was certified at a certain number of steps, and the students were rewarded with a certificate indicating their achieved level (Figure 1).

### 2.2 Test environments

The general certification procedure (Figure 1) was tested in various courses at both bachelor and master level in biology and geology, which had in common that the practical use of light microscopy was an ILO. To evaluate the efficiency of the certification procedure across different learning environments, data were collected in 2021 and 2022 at three different academic institutions in Norway (University of Bergen (UiB), the University Centre in Svalbard (UNIS), and the University of Oslo (UiO)). At UiO, the protocol was slightly adjusted between years based on feedback collected the first year (Table 1).

### 2.3 Certification procedure and data collection at UNIS

At UNIS, a certification procedure with three levels was developed (Table 2). The aim at UNIS was to use the certification procedure as a mandatory activity in all courses planning to use microscopes. Besides improved alignment, another motivation to implement certification for all users was to reduce the misuse of microscopes and prevent damage to expensive equipment.

**TABLE 2:** Example of a three‐step certification procedure used with Leica DM750 light microscope at the University Centre in Svalbard.

Assignment 1. Name all the parts of a microscope	Assignment 2. Set up a microscope for work	Assignment 3. Examine a slide underneath the microscope
Students sit with a microscope, points to parts and explains. Students must name, point to and explain what the listed parts do, in order to be certified for this part. Our tick off list:	Students are given a ready slide (needed to adjust eyepieces). The task is to set up the microscope from unpacked microscope to ‘ready to go’, with slide in position, and then go back to packed microscope. Our tick off list:	Students are given a ready slide. The task is to have a diatom in focus at 40× magnification, use phase contrast at 40× and then take the slide away again. They need to be able to show all the steps in order to be certified. Our tick off list:
Eyepiece: adjust scale Tubes: adjust distance between eyes Carrying handle: to carry microscope (both hands!) Objectives: magnify, how to calculate magnification, what about use of 100× Stage: changes position of slide, markers Focus: coarse and fine, use coarse only at 10× in beginning Power: switch on Illumination: from below Condenser: Function, why and what does it Phase contrast: Function, why and what does it Nosepiece: how to rotate objectives	Switch the light on Adjust the chair height Adjust the distance between eyes Adjust the eyepiece (if possible) Put slide on stage (they must know only to start with 10× magnification, and to put the stage in low position) Then dismantle everything again (do not forget to switch off the light)	They have to put the stage in low position They have to start with the smallest magnification 10× Then work with 20× (put in focus) And only then go to 40×, and focus Use phase contrast on 40× From 40× they need to go to 20×, then to 10x (DO NOT rotate past 100×! but rotate back) Put stage in low position

The certification procedure was accomplished during a 2‐h lab session (the introduction to the microscopes, the practice part, and the full certification procedure). From March 2021 until June 2022, the certification procedure was performed within eight courses, with a maximum of 20 students attending one session (Table 1).

A survey was created in nettskjema (nettskjema.no), and all attendees were asked to answer the survey afterwards. The survey contained 18 questions with three to five alternative answers (17 regarding various aspects of the certification procedure; Appendix S1; and one question regarding the survey itself). The survey was anonymous, and the only data collected about the responders were whether they were students or staff and their skill level in using a light microscope prior to the exercise (with alternatives; Highly skilled/Skilled/Neutral/Less skilled/No experience from before). The survey was open from March 2021 to June 2022, covering the period from when the first course was given and closed 2 weeks after the last course. As the survey remained open continuously during the sampling period, it was not possible to divide responses into courses.

### 2.4 Certification procedure and data collection at UiB

The same certification procedure as used at UNIS was tested in one course at UiB with 15 students in 2021 and again in 2022. The feedback was provided orally to the teacher. We did not have appropriate approval for detailed data collection, so the feedback presented is a summary provided by the instructor and represents a general impression.

### 2.5 Certification procedure and data collection at UiO

At UiO, the certification protocol was slightly adjusted to fit a different microscope, with three (2022) or four (2021) levels developed for a first‐year course in biology as one of their 2‐h lab exercises (Table 1). The certification procedure has been run twice in this course (autumn 2021 and autumn 2022), with some adjustments between years.

In contrast to UNIS, the students had to pass the certification to sit the exam in the course. Further differences were in the level of preparation. The preparation lecture for 2021 was held the week before, and the protocol was provided beforehand. It was emphasized that the students needed to prepare before the lab exercise, as they had to pass the certification to sit the exam. However, as we tested the certification procedure for the first time, the stake in the activity was reduced by informing us about a second possibility to pass the certification 2 weeks later (although no one needed this in the end). As more time was available in the lab, an additional level of certification (regarding Köhler illumination) was added to the certification procedure. Also, the person certifying the level had to sign the certification protocol for this level. The intention of including this step was to increase the feeling of obligation. A certificate stating the achieved skill level was distributed after passing the certification. The students needed to pass three of four levels to get a certificate and sit the exam.

In 2022, the introduction lecture was shortened to about 10 min, only focusing on how the certification procedure should be performed. The actual introduction to the microscope was done by students preparing themselves by reading a lab manual and watching a set of instruction videos prepared for this purpose (LINK/IBV, 2022). The first step of certification from 2021 (focusing on naming parts and functions of the microscope) was taken out of the actual lab session and instead tested beforehand through a mandatory quiz created in the learning management system CANVAS. Students were required to score at least 10 out of 12 on the quiz to be allowed to participate in the lab. The students could redo the quiz as many times as they wanted, but the right answers were not revealed if they failed the quiz. This was to force students to prepare, (re‐) watch the videos, and read the compendium to find the answers. The certification in the lab was then reduced to three steps.

Surveys were created in nettskjema (nettskjema.no) and distributed to all students after the lab sessions, and was open for 3 weeks. In 2021, the survey included 12 questions graded on the Likert scale (Appendix S2), one item with a yes/no option (‘I certified others’), and the question ‘I prepared for the lab exercise’ (No/Partly/Yes). As the preparation part was changed from 2021 to 2022, the survey was extended in 2022 to include 21 questions graded on the Likert scale (11 were kept, 1 removed, 10 were added; Appendix S2) and one item with a yes/no option (“I certified others”). Items with graded Likert scales were rescored as: Fully disagree = 1, Disagree = 2, Neutral = 3, Agree = 4, Fully agree = 5. Both surveys included a free text possibility at the end with ‘Other comments/suggestions’. The survey was anonymous, and the only data collected about the responders were their skill level in using a light microscope prior to the exercise.

### 2.6 Data handling

All survey data were summarized as distributions in counts and percentages among alternative answers per question. The data were also summarized separately based on skill level to evaluate differences. As data from UNIS were collected from various courses, the data were plotted towards time to check for clustered outliers. No such trends were detected. Four questions (question 1, 9,10, and 12; Appendix S1) with an ordinal ranking from UNIS were coded 1 to 5 (e. g. when the five alternatives differed, like 1 – significantly less, 2 – slightly less, 3 – neither less nor more, 4 – slightly more, and 5 – significantly more) in order to visualize the responses similar to a Likert scale to ease comparison with the surveys from UiO.

The coded data from UNIS and the data from UiO collected at the Likert scale were summarized and plotted with the Likert function included in the HH v.3.1 package run in R v 4.2.1 (Heiberger, 2020; R Core Team, 2020). We analysed the ranked Likert scale (1–5) by standard descriptive statistics in R v 4.2.1 and PAST 4.08 (Hammer et al., 2001), including median score per question with 25th and 75th percentiles calculated, and non‐parametric statistic tests. The Kruskal‐Wallis test was used to test for equal medians between years and among skill levels, and a Mann‐Whitney U test was used for pairwise comparisons with and without Bonferroni correction.

At UiB, experiences were collected orally after the certification, and the feedback noted and summarized as a general evaluation by the course leader. No personal data was noted, and the impression presented here is the general impression from the course leader who is a part of the project.

## 3 RESULTS

We obtained 86 survey responses from UNIS (Appendix S1), representing at least 50% of the potential pool of responders. The survey included one question about the perception of the survey itself: 38% found all questions easy to understand, 36% found one or two questions a bit difficult, whereas 26% found the survey difficult in general. We obtained survey responses from about 50% of the potential pool of responders at UiO (51 in 2021, 70 in 2022; Appendix S2). About 80% of the responding students contributed to certifying others (41 of 51 in 2021; 56 of 70 in 2022).

### 3.1 Skill level prior to the exercise – All institutions

There was a clear difference in reported skill level prior to the exercise among institutions. At UiB, the students were at least 2nd year bachelor students, and based on oral feedback, the students expressed that they already felt skilled in microscopy and that the level of the certification was somewhat below their initial skill level. At UNIS, participants were a mix of second‐year bachelor students and staff members, and 64% considered themselves Skilled or Highly skilled (*n* = 45 and 10 respectively) before the certification, and only three responders reported being less skilled or having no experience from before. The rest were neutral (*n* = 28). Thus, at UNIS, medians were only compared among those reported to be Neutral, Skilled, or Highly Skilled for the four questions coded to a Likert scale. At UiO, the participants were following a first‐year bachelor course, and only 20% (2021) and 13.7% (2021) considered themselves Skilled or Highly skilled before. The majority reported being less skilled or having no experience from before (51% in 2021; 62.7% in 2022).

### 3.2 The University Centre in Svalbard ‐ UNIS

**FIGURE 2:**
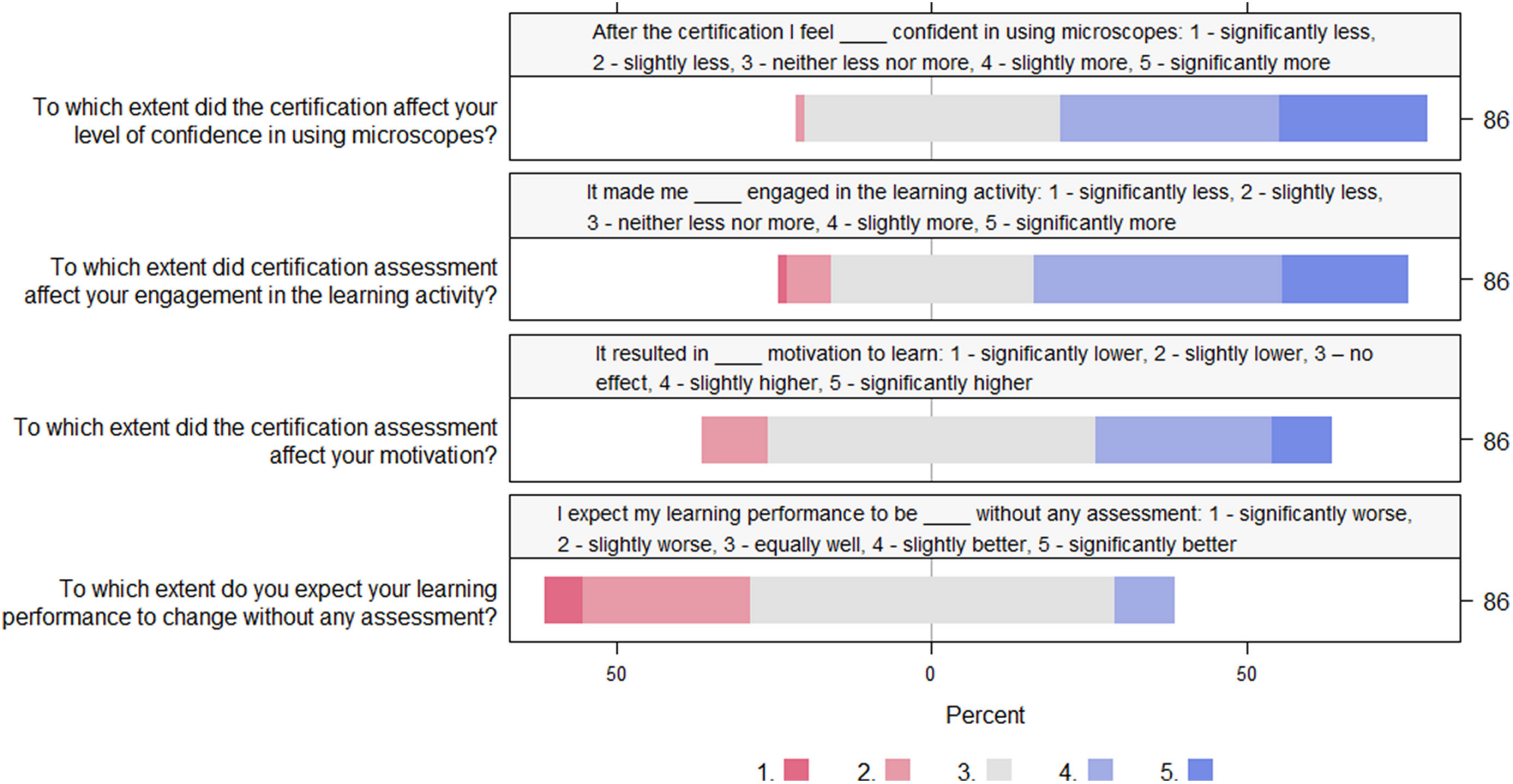
Combined survey responses collected after eight iterations of practical introduction to light microscopy using a certification procedure at the University Centre in Svalbard. The diverging stacked bar chart show the percentage of each response category to statements from four questions from the survey (questions 1, 9, 10, and 12; Appendix S1) with an ordinal ranking coded 1 to 5 in order to visualize the responses. Number of respondents given to the right.

At UNIS, 57% reported being more confident in using a light microscope after the certification procedure, but for this statement there was a significant difference among skill levels (Kruskal‐Wallis *χ* ^2^ = 15.473, df = 2, *p*‐value < .001). Those being ‘Highly skilled’ from before were neutral to this question. A majority expected their learning outcome to be similar without any certification, but 33% reported they expected to do worse without the assessment (Figure 2).

The certification procedure made students more engaged in the activity; 60% reported being slightly more or significantly more engaged, and there was a skew towards higher motivation to learn (52% neutral, 32% higher motivation; Figure 2). For this statement there was also a significant difference among skill levels (Kruskal‐Wallis *χ* ^2^ = 8.3899, df = 2, *p*‐value < .05). The motivation of those being ‘Highly skilled’ was not affected by the certification procedure.

The awareness of being certified did in general not affect student's emotions (70% were neutral), but students felt they learned something during the procedure (67% learned something, 14% learned a lot), and for some, the depth of what they learned was slightly increased and that the scope was slightly widened (Questions 6–8, 11; Appendix S1).

The experienced risk of failing the certification was low (98% reported quite unlikely or very unlikely to fail), but there was no wish for this to be changed. Introducing grading rather than pass/fail to increase motivation was not supported; a clear majority would not be more motivated if it was graded (71% was neutral or expected to be less motivated if the procedure was graded). The certificate provided after passing the procedure was regarded as piece of paper of little value (Questions 2–5, 13; Appendix S1).

There was no wish to change or terminate the certification procedure; 95% wanted to keep the certification procedure, and 59% reported that the certification procedure should remain the same. A slight majority (55%) supported introducing certification to test other practical skills as well (Questions 14–17; Appendix S1).

### 3.3 University of Bergen ‐ UiB

At UiB, the general feedback was that the certification procedure was fine but a bit superfluous. The course the certification procedure was tested in was on an advanced bachelor level, and the students experienced already being at the targeted skill level. The students in 2021 had less experience using microscopes compared to the students in 2022. In both years, the students saw the potential in the method and liked to get refreshed before they started their own laboratory work, which includes the use of microscopes. During the oral feedback, students were asked to suggest improvements to the protocol. Several suggested to exchange the oral instruction from the teacher with an instructional video that could be watched at home before coming to the lab.

### 3.4 University of Oslo ‐ UiO

Our results showed that the certification procedure itself increased the level of preparation to the exercise. In the first iteration in 2021, there were no mandatory activities before the lab. Still, most students prepared for the lab exercise; 73% answered ‘Yes’ and 27% ‘Partly’ to ‘I prepared for the lab exercise’, and 55% reported that the certification procedure made them prepare more than usual (Appendix S2).

**FIGURE 3:**
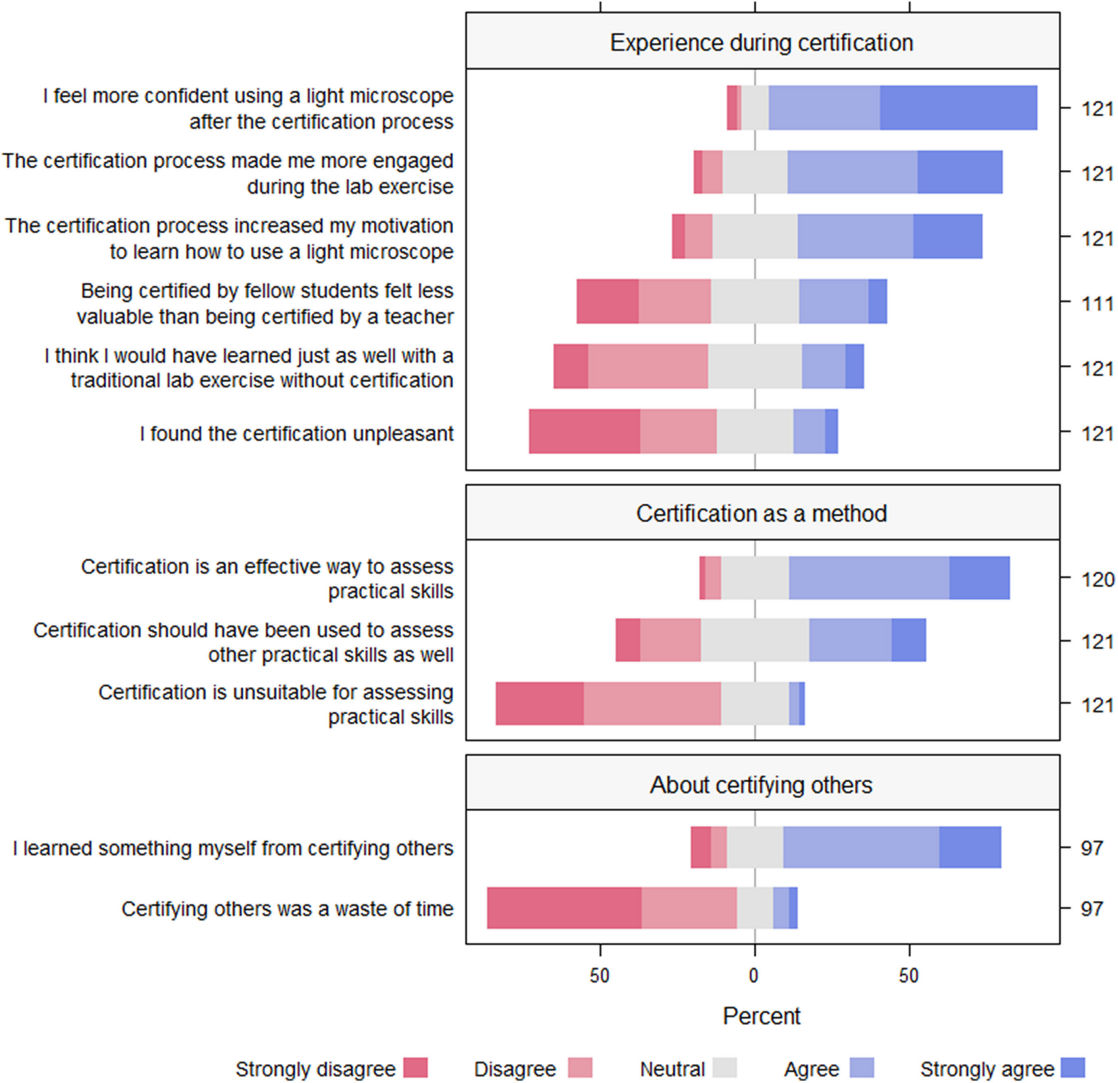
Combined survey responses collected after two iterations (51 in 2021 and 70 in 2022) of practical introduction to light microscopy using a certification procedure at the University of Oslo. The diverging stacked bar chart show the percentage of each response category to statements from 11 questions included in both surveys. Number of respondents given to the right. Although the procedure was somewhat adjusted between years, there were no significant differences in how the students responded between years (Figure S1; Appendix S2).

As the introduction to the lab exercise was changed and mandatory tasks were added between years, questions regarding preparation were adjusted and expanded in the 2022 iteration and presented in more detail below. The remaining 11 survey questions from the iteration in 2021 (about students' experiences during certification, certifying others, and certification as a method) were also part of the survey after the iteration in 2022. There were no significant differences in median scores for these questions between years, and the distribution along the Likert scale was highly congruent (Figure S1; Appendix S2). Thus, we combined the results from 2021 with the survey results from 2022 (Figure 3).

The results clearly showed that the certification procedure made students more confident in using a light microscope (87% agree or strongly agree; experience during certification; Figure 3). In addition, the certification made 69% of students more engaged in the lab exercise, and 60% reported that it increased their motivation to learn how to use a light microscope. Half of the students (50%) disagreed or strongly disagreed with the statement, ‘I think I would have learned just as well with a traditional lab exercise without certification’ (Experience during certification; Figure 3.).

Certification was regarded as an efficient method to assess practical skills (Certification as a method; Figure 3). A similar statement, ‘Certification is a good way to learn practical skills’, was only included in 2022, but more than 70% agreed or strongly agreed with this statement (Appendix S2). Interestingly, the only statement showing a close to significant difference in response distribution between 2021 and 2022 was ‘Certification should have been used to assess other practical skills as well’. Students showed an overall neutral response (Certification as a method; Figure 3), but were somewhat more positive towards implementing certification for other practical skills in 2022 than in 2021 (Figure S1; Appendix S2). Our results further showed that certifying others increased the perceived learning outcome (about certifying others; Figure 3).

Twelve comments were collected through the free text option in 2021, whereof nine stated that they liked the certification procedure. One mentioned that it created variation, and another found it more motivating for learning. In six of the 12 comments, it was mentioned that there was too little time for the certification procedure. Three mentioned that it was a bit unpleasant or difficult to certify others, in particular when they failed. One mentioned that it was hard to know if the student they were supposed to certify actually saw what they were supposed to see in the microscope. One student experienced the certification as a total failure, as the peers that had certified this student provided the wrong instructions, and the student had learned it all wrong. One suggestion for improvements was to make instructional videos as preparation material.

**FIGURE 4:**
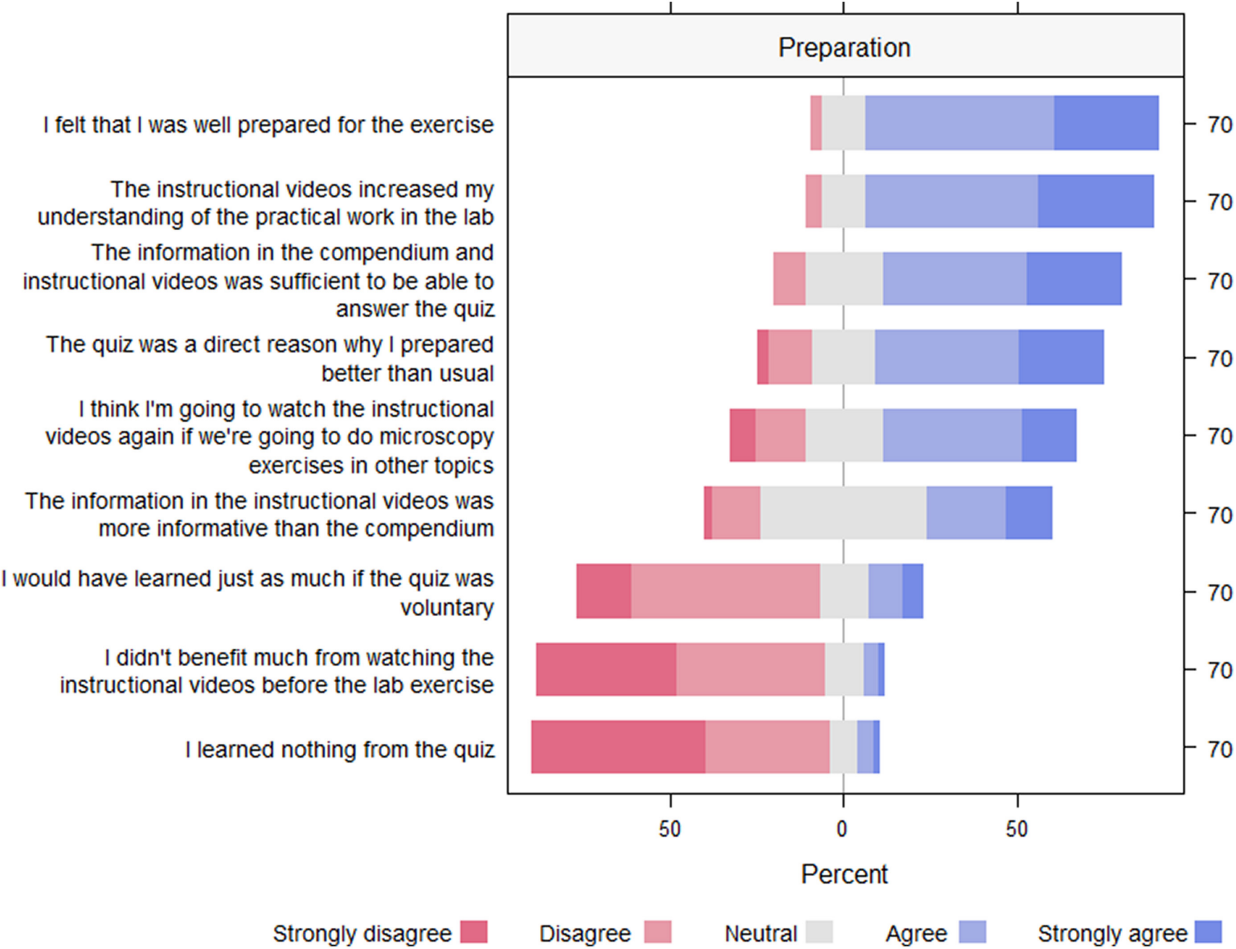
Combined survey responses collected after two iterations (51 in 2021 and 70 in 2022) of practical introduction to light microscopy using a certification assessment at the University of Oslo. The diverging stacked bar chart shows the percentage of each response category to statements from 11 questions included in both surveys. The number of respondents given to the right. Although the procedure was somewhat adjusted between years, there were no significant differences in how the students responded between years (Figure S1; Appendix S2).

Based on this feedback, we adjusted the iteration in 2022 as explained in the material and methods section; instruction videos were made and included, the number of certification steps in the lab was reduced to provide more time, and the first step of the certification was instead evaluated through a mandatory pre‐lab quiz. As the preparation part was changed and extended in 2022, we added a set of additional questions to the survey in 2022. The response showed that the quiz and the instruction video clearly made the students feel prepared for the exercise (Figure 4). The response also showed that the students doubted they would learn equally well if the quiz was not mandatory (Figure 4).

At UiO, medians were compared among the five skill levels: ‘No experience from before’ (*n* = 27), ‘Less skilled’ (*n* = 41), ‘Neutral’ (*n* = 32), ‘Skilled’ (*n* = 14), or ‘Highly Skilled’ (*n* = 7). Two statements showed significant differences among different skill levels. Those with ‘No experience’ with light microscopy found the instructional videos more useful than those being ‘Highly skilled’ (Kruskal‐Wallis *χ* ^2^ = 11.667, df = 4, *p*‐value < .01), and they were also more positive to watch them again later (Kruskal‐Wallis *χ* ^2^ = 10.261, df = 4, *p*‐value < .05), but the pairwise comparison was not significant after Bonferroni correction.

We found that there were some technical issues with the quiz and some questions that could be misunderstood, which created frustration. We received 16 free text comments in 2022, and most of them pointed out that the quiz should be improved, and several of the responders again raised lack of time as an issue. Two students questioned whether more mandatory work would improve their motivation for learning. Interestingly, some suggested exchanging instruction videos with a discussion with their partner in front of the microscope, that is, changing it back to the original protocol. It was also pointed out that the assessment and certification should be done by teachers rather than students. On the other side, several mentioned that they learned a lot, and it was commented that the certification procedure made sure that they all learned everything rather than delegating tasks among them.

## 4 DISCUSSION

Regardless of institution, type of preparation material, and level of former experience, the certification procedure made students more engaged in the exercise, and the majority reported that the certification increased their motivation to learn, increased their perceived learning outcome, and that the certification procedure was regarded as appropriate for assessing practical skills. The type of certification procedure developed and tested in this study is an efficient way of doing a direct assessment of practical lab skills.

‘I find this much more motivating for learning, because often in labs we often just focus on getting the given task done…’ open answer from student survey.

### 4.1 Increased constructive alignment do enhance engagement and improve learning

Our results strongly support Biggs' constructive alignment as a powerful teaching principle enhancing learning. The fact that the certification procedure created higher engagement and motivation (Figures 2 and 3, oral feedback UiB) suggests that we successfully managed to design a learning activity that activated students in their own learning and helped them reach the intended learning outcomes (ILOs). Students became more confident in using a light microscope (57% at UNIS and 93% at UiO agreed or strongly agreed; Figures 2 and 3). The feedback further supported the link between ILOs, learning, and aligned assessment, as the majority reported they would have learned less without the integrated assessment through certification.

The way constructive alignment puts ILOs up front can be problematic and has been criticized (Hussey & Smith, 2002). The criticism is partly connected to the way constructive alignment has been utilized by policy makers and academic administrators (Loughlin et al., 2021). By becoming a part of for example, quality assurance systems, constructive alignment has to some extent drifted away from its original meaning and into an administrative exercise needed for accountability purposes (Loughlin et al., 2021). We do not advocate that *all* ILOs can or should be directly assessed, but our results provide support for former findings showing that when constructive alignment is used in the way it was intended, it enhances learning (Adams, 2020; Loughlin et al., 2021).

### 4.2 Instruction videos and mandatory quizzes – Beneficial but not essential

Former studies have shown that using instruction videos in combination with pre‐quizzes for lab preparation increases both lab efficiency and learning outcomes (Croker et al., 2010; Onyeaka et al., 2022). Access to instruction videos and more time to practice were identified as possible improvements to the certification procedure at both UiB and UiO. Thus, as a response to this feedback, the preparation procedure at UiO was changed from an introductory lecture in 2021 (not mandatory) to self‐study based on reading material, instruction videos, and a mandatory quiz in 2022. The quiz covered the names and functions of the microscope and represented level 1 of the certification procedure that was done in situ the year before (as in UNIS; Table 2). This change released more time to practice other skills during the lab session. The students reported that the quiz and the instruction videos increased their understanding and made them more prepared, supporting the idea that investment in instruction videos provides students with useful tools for preparation and repetition (Croker et al., 2010; Müller et al., 2019; Onyeaka et al., 2022). However, the availability of instruction videos and mandatory preparation did not change the level of engagement, motivation, or perceived learning outcome between years. Thus, the certification procedure per se, including tactile activities with the microscope, was the most significant factor in improving these crucial aspects of learning. However, the risk of failing was higher at UiO than at the other institutions (discussed further below). The majority at UiO reported preparing more than usual for the lab session in 2021, even if it was not mandatory. Thus, the risk of failing the certification procedure may have motivated more preparation.

### 4.3 The risk of failing influences efficiency

The risk of failing differed between institutions. At UiO, the students had to pass the certification to sit the exam (it was part of the summative assessment), whereas at UNIS and UiB there were no real consequences of failing (it was purely formative). This might partly explain why the overall engagement was higher at UiO than at UNIS and that the level of preparation was high at UiO. Students are more likely to prioritize learning outcomes that are assessed and prioritize activities after the level of importance, that is, the backwash effect (Biggs & Tang, 2011; Elton, 1987; Hattie, 2015; Watkins et al., 2005). It is clear that assessment can both enhance and hinder learning, depending on how the assessment is designed and implemented in a given learning environment (Yan & Boud, 2022). Assessments related to ‘high‐stake’ can, for example, be contra‐productive for learning, even when the assessment is well‐aligned with the ILOs (Raaheim, 2019). Higher levels of anxiety for high‐stake tests result in, for example, less retention of knowledge (Hinze & Rapp, 2014). This is important to take into account when planning a certification procedure.

Although most students felt comfortable being certified by peers, some students expressed concern about peers making mistakes. There are challenges related to how much assessment responsibility one can place on students, but the benefits of peer assessment, peer instruction and peer review among students have been thoroughly supported empirically (Crouch & Mazur, 2001; Harland et al., 2017; Harris, 2011; Reddy et al., 2021). Students learn, and they usually feel safe as the power balance is even. Our results also show that students learned from certifying others (Figure 3). However, lack of power balance can also hamper learning when peers try to avoid revealing being uncertain or having knowledge gaps (Ladyshewsky, 2013). Being aware of this risk, we suggest constructing ways to mitigate such situations. One idea could be to introduce the possibility for asking for a ‘second opinion’, that is, a sign certifiers can use to call for help from another certifier or instructor if they are unsecure. To make this work, instructors must make it clear from the beginning that it is normal that recently certified students feel unsecure and that it is fully acceptable to ask for help if needed. Another option could be random controls by a teacher, checking that the peer assessment is up to standards.

### 4.4 Initial skill levels and heterogeneous student groups influence efficiency

Besides differences in risk of failing, the initial skill levels clearly differed between institutions. Our results emphasize the importance of tuning the level of difficulty to the expected pre‐knowledge. Comparing results between UiO (less skilled) and UNIS (more skilled), showed that students with no or little experience in microscopy before the exercise were overall more positive about the certification procedure compared to skilled students, and experienced a higher learning outcome. For example, our results show that the certification procedure increased motivation overall at both UNIS and UiO (Figures 2 and 3). However, those reporting being ‘Highly skilled’ at UNIS did not become more motivated by the certification procedure. More skilled students experienced a lower learning outcome. This is not surprising, as our protocols were aligned towards less skilled students; all three certification protocols assumed no former knowledge. Why the ‘Highly skilled’ reported no effect on motivation is probably related to the lack of challenge. Yan and Boud (2022) claim that to create an assessment‐as‐learning situation, the assessment must represent some challenge to promote deeper learning, and we did clearly not provide an appropriate challenge for the highly skilled students. An extra set of skill level asking for a more advanced skill set might be an option in heterogeneous student groups. Such differentiation should however only be done in the last step of the certification procedure, otherwise it will be difficult to efficiently use peer‐assessment.

Alternatively, it might be better to provide more support up front to level out the differences. Both pre‐quizzes and peer reviews have been advocated as strategies to buffer heterogeneity among students in lab courses (Schäfer & Brück, 2013). Our results showed that unexperienced students found the instruction videos significantly more useful than more experienced students, which suggests that instruction videos can also contribute to buffering initial differences in skill levels.

### 4.5 Do it yourself

At UiO, the students regarded the certification procedure efficient to assess practical skills, and at both UNIS and UiO the majority was positive to use certification procedures to assess other practical skills as well. Thus, the described certification procedure seems robust and can easily be adjusted and used in assessing other practical skills. Based on our experience, we have tried to synthesize a ‘best practice’ for certification of practical skills.

1. The tick‐off list should specify the learning outcomes, which topics should be conveyed, and which information should be included. List the following information in accordance with the level of importance (starting from the most essential): desired practical and theoretical learning outcomes to be assessed, all other topics that are important to convey, other information that the learner should be exposed to during the certification.
2. Decide on the level of impact the certification should have. How important is it to pass? Should it be graded?
3. Divide your list in appropriate skill‐levels if you plan for several levels
4. All students should be given the possibility to practice/prepare themselves before the certification procedure.
5. You should think about how much time you would like to allocate for this. Some procedures will be more work‐demanding than others and should be aligned with the importance of the specific learning activity to reach the overall ILOs of the course.
6. Based on this information, make a protocol for certification (a tick‐off list; Table 2), and make sure that the students are given learning activities that align with the certification (that they practice the skills that later on are assessed). Try to include the points you rank on top.
7. Take into account student heterogeneity, initial skill level, and whether the students know each other. Peer assessment will work best when students feel safe and trust each other, so avoid doing this type of exercises at day 1 of a course.

We did not collect data that evaluated the gaming elements of our procedure, but we observed that it was important to the students to physically receive the post‐it note with the right colour when passing a level (even though it was the signature in their protocol that actually mattered for getting the certificate in the end). Experiences from gamification have shown that this type of badge triggers motivation to learn (Bai et al., 2020; Rutledge et al., 2018). If possible, we suggest adding some visible symbol for passing skill levels during certification.

We experienced that students did prepare better for the exercise and claimed to learn more when elements of summative assessment were blended in. However, we suggest including certification procedures as a ‘low‐stake’ activity when you are testing a new protocol for the first time. When you know the protocol works well, we suggest adjusting the certification procedure towards an assessment‐as‐learning procedure blending formative and summative assessment.

Yan and Boud (2022) defined assessment‐as‐learning as ‘Assessment that necessarily generates learning opportunities for students through their active engagement in seeking, interrelating, and using evidence’. In their view, assessment‐as‐learning should contain three core elements: (1) the purpose is to promote learning as well as evaluating students' performance; (2) it requires students to learn from engagement with the assessment task itself as well as activities associated with it; and (3) it requires students to take an active and reflective role and thus foster metacognition and self‐regulation (Yan & Boud, 2022). Our certification procedure clearly meets core elements 1 and 2, but a more explicit reflective part should be added. The surveys distributed in connection with the activity forced students to reflect on their learning, but for the future, this should be integrated as a natural part of the certification procedure. This is something we are currently integrating and testing further.

### 4.6 Skills on a diploma?

The certificates we provided did not seem to represent any particular value for the students. They were not recognized in a quality system where, for example, employers or supervisors could evaluate the credibility of this certificate. Many universities have already implemented various forms of digital badges or micro‐credentials as an add‐on to their ordinary graded subjects, and this trend towards micro‐credentials and skill‐competency‐based training is growing globally (McGreal & Olcott, 2022). According to McGreal and Olcott (2022), micro‐credentials are ‘certified documents that provide recognized proofs of the achievement of learning outcomes from shorter, less duration, educational or training activities’. These are currently non‐credit achievements, and not integrated in ordinary university programmes. We will encourage university leaders looking into the possibilities of implementing similar types of, for example, skill certificates or micro‐credentials related to discipline skills as part of degree‐programmes, with potential repetition and advancement during the study programme.

## 5 CONCLUSION

The presented certification procedure is appropriate and efficient for both learning and assessing practical microscopy skills. The procedure increased alignment and enhanced perceived learning. The presented certification procedure can easily be adapted to assess other practical skills and can, with some adjustments, be an efficient method for assessment‐as‐learning, merging formative and summative assessment. It is, however, important to account for student heterogeneity and adjust the level of difficulty to the initial skill level. Peer assessment made the procedure efficient, but it work best when students feel safe and trust each other.

## AUTHOR CONTRIBUTIONS

**Pernille Bronken Eidesen:** Conceptualization (lead); formal analysis (lead); funding acquisition (lead); investigation (equal); methodology (equal); project administration (lead); resources (equal); supervision (equal); validation (equal); visualization (lead); writing – original draft (lead); writing – review and editing (equal). **Anne E. Bjune:** Investigation (equal); resources (equal); validation (equal); writing – review and editing (equal). **Simone I. Lang:** Conceptualization (supporting); investigation (equal); methodology (equal); project administration (supporting); resources (equal); supervision (equal); validation (equal); writing – review and editing (equal).

## Supporting information


Appendix S1‐S2

Figure S1

## Data Availability

All participants were informed and consented that the data could be used for scientific purposes. No personal data have been collected. The appendices represent a full overview of the data, and the original data matrices allowing linking answers across questions are available through Centre for Open Science (https://osf.io/8ykdr/?view_only=384c820873bd477bb67b13bbc044b2f6).
