# Supplementary material for: “Show me how to use a microscope” – The development and evaluation of certification as direct assessment of practical lab skills
Source: Ecol Evol. 2023 Oct 11;13(10):e10592. doi: 10.1002/ece3.10592 (PMC10568202; doi:10.1002/ece3.10592)
Supplement: Supplementary file 1 — Appendix S1‐S2 [file ECE3-13-e10592-s001.docx]

**Appendix 1.** Survey responses after performing a 2-h introduction and certification procedure in light microscopy at the University Centre in Svalbard in eight different courses during the period March 2021 - June 2022, with 10-20 participants per course, 86 responses were collected cumulatively in the period. N – counts, % - percentage.

| **Questions and alternatives** | **N** | ***%*** |
| --- | --- | --- |
| *1. To which extent did certification assessment affect your engagement in the learning activity?* | | |
| It made me significantly more engaged in the learning activity. | 17 | *20* |
| It made me slightly more engaged in the learning activity. | 34 | *40* |
| It made me neither less nor more engaged in the learning activity. | 28 | *33* |
| It made me slightly less engaged in the learning activity. | 6 | *7* |
| It made me significantly less engaged in the learning activity. | 1 | *1* |
| *2. To which extent would your motivation change if the certification assessment was graded?* | | |
| I would be significantly more motivated. | 5 | *6* |
| I would be slightly more motivated. | 15 | *17* |
| I would be neither less nor more motivated. | 44 | *51* |
| I would be slightly less motivated. | 9 | *10* |
| I would be significantly less motivated. | 13 | *15* |
| 3. What do you think about the likelihood of failing the certification assessment? | |  |
| It was very likely to fail the certification assessment. | 0 |  |
| It was quite likely to fail the certification assessment. | 0 |  |
| It was reasonably likely to fail the certification assessment. | 2 | *2* |
| It was quite unlikely to fail the certification assessment. | 34 | *40* |
| It was very unlikely to fail the certification assessment. | 50 | *58* |
| *4. To which extent would the likelihood of failing the certification assessment affect your opinion on whether the certification assessment is worth spending time on (including time needed for preparation)?* | | |
| If the likelihood to fail the certification assessment would be higher than it was, it would be more worth to spend time on. | 16 | *19* |
| The likelihood of failing the certification assessment does not affect my opinion on whether the certification assessment is worth spending time on. | 66 | *77* |
| If the likelihood to fail the certification assessment would be lower than it was, it would be more worth to spend time on. | 4 | *5* |
| *5. To which extent should the likelihood of failing the certification assessment be changed?* | | |
| It should be significantly increased. | 1 | *1* |
| It should be slightly increased. | 2 | *2* |
| It should not be changed. | 80 | *93* |
| It should be slightly decreased. | 2 | *2* |
| It should be significantly decreased. | 1 | *1* |
| *6. To which extent did your awareness about the certification assessment affect the scope of what you learn during the course?* | | |
| The scope of what I learn becomes significantly wider compared to what I would have learned otherwise. | 6 | *7* |
| The scope of what I learn becomes slightly wider compared to what I would have learned otherwise. | 28 | *33* |
| Me being informed about the certification assessment has nothing to do with the scope of my learning activity during the course. | 51 | *59* |
| The scope of what I learn becomes slightly narrower compared to what I would have learned otherwise. | 1 | *1* |
| The scope of what I learn becomes significantly narrower compared to what I would have learned otherwise. | 0 |  |
| *7. To which extent did your awareness about the certification assessment affect the depth of what you learn during the course?* | | |
| The depth of what I learn becomes significantly deeper compared to what I would have learned otherwise. | 3 | *3* |
| The depth of what I learn becomes slightly deeper compared to what I would have learned otherwise. | 20 | *23* |
| Me being informed about the certification assessment has nothing to do with the depth of my learning activity during the course. | 59 | *69* |
| The depth of what I learn becomes slightly less deep to what I would have learned otherwise. | 3 | *3* |
| The depth of what I learn becomes significantly less deep compared to what I would have learned otherwise. | 1 | *1* |
| *8. To which extent did the certification procedure itself affect what you learned?* | |  |
| I learned a lot during the certification procedure. | 14 | *16* |
| I learned something during the certification procedure. | 58 | *67* |
| I learned nothing during the certification procedure. | 14 | *16* |

| *9. To which extent did the certification affect your level of confidence in using microscopes?* | | |
| --- | --- | --- |
| After the certification I feel significantly more confident in using microscopes. | 20 | *23* |
| After the certification I feel slightly more confident in using microscopes. | 30 | *35* |
| It had no effect on my confidence level in using microscopes. | 35 | *41* |
| After the certification I feel slightly less confident in using microscopes. | 1 | *1* |
| After the certification I feel significantly less confident in using microscopes. | 0 |  |
| *10 To which extent do you expect your learning performance to change without any assessment?* | | |
| I expect my learning performance to be significantly better without any assessment. | 0 | *5* |
| I expect my learning performance to be slightly better without any assessment. | 8 | *9* |
| I expect to learn equally well without any assessment. | 50 | *58* |
| I expect my learning performance to be slightly worse without any assessment. | 23 | *27* |
| I expect my learning performance to be significantly worse without any assessment. | 5 | *6* |
| *11 To which extent did the certification assessment affect your emotions?* |  |  |
| It created significant excitement making learning more pleasant. | 2 | *2* |
| It created slight excitement making learning more pleasant. | 19 | *22* |
| It was not affecting my emotions. | 60 | *70* |
| It created slight psychological pressure making learning less pleasant. | 5 | *6* |
| It created significantly psychological pressure making learning less pleasant. | 0 | *1* |
| *12. To which extent did the certification assessment affect your motivation?* |  |  |
| It resulted in significantly higher motivation to learn. | 8 | *9* |
| It resulted in slightly higher motivation to learn. | 24 | *28* |
| It had no effect on motivation. | 45 | *52* |
| It resulted in slightly lower motivation to learn. | 9 | *10* |
| It resulted in significantly lower motivation to learn. | 0 | *1* |
| *13. To which extent is the certificate itself (the document that you are awarded with) somehow useful to you?* | | |
| The certificate itself is very useful to me. | 2 | *2* |
| The certificate itself is quite useful to me. | 16 | *19* |
| I do not understand yet whether the certificate itself can be useful to me. | 28 | *33* |
| The certificate itself is rather useless to me. | 19 | *22* |
| The certificate itself is totally useless to me. | 21 | *24* |
| *14. What approach to certification procedure is better?* |  |  |
| The certification procedure should have several levels/assignments compulsory for everyone. | 14 | *16* |
| The certification procedure should have only one level/assignment. | 22 | *26* |
| The certification procedure should have several levels/assignments with freedom to choose one option. | 8 | *9* |
| The certification procedure should have several levels/assignments with freedom to combine several options. | 4 | *5* |
| I do not think that any of these approaches is better than others. | 38 | *44* |
| *15. What purpose the certificate itself should serve?* |  |  |
| It should confirm that a person awarded with the certificate got some qualifications and abilities that may be different for different people. | 22 | *26* |
| It should confirm that a person got a standard minimum of competence plus some advanced (personalized) qualifications and abilities. | 17 | *20* |
| It should confirm that each person awarded with the certificate got standard minimum of competence same as everyone else. | 44 | *51* |
| It should serve mainly ceremonial purposes and symbolize public recognition of efforts invested into learning. | 3 | *3* |
| *16. What do you think about the future of this certification assessment procedure?* | |  |
| This certification procedure should remain the same. | 51 | *59* |
| This certification procedure should not be terminated, but it should be changed/improved. | 28 | *33* |
| This certification assessment procedure should be terminated. | 7 | *8* |
| *17. What do you think about the future of certification procedures at UNIS in general (including certification procedures in other settings, for example, for testing practical field skills)?* | | |
| This certification procedure should not be terminated and certification procedures in other settings should also be introduced at UNIS. | 47 | *55* |
| This certification procedure should not be terminated, but certification procedures in other settings should not be introduced at UNIS. | 34 | *40* |
| This certification procedure should be terminated and certification procedures in other settings should not be introduced at UNIS. | 5 | *6* |

**Appendix 2.** Survey responses (51 in 2021, 70 in 2022) after performing a lab exercise with certification procedure in light microscopy at the University of Oslo in a first year biology course in 2021, and again in 2022 after making adjustments based on feedback from 2021. The level of agreement with the given statements was graded after a five-point Likert scale: 1 – strongly disagree, 2 – disagree, 3 – neutral, 4 – agree, 5 – strongly agree. In addition, there was a yes/no question asking whether they certified others or not. M-median, 25 - 25 percentile, 75 -75 percentile.

| **Question/statement** | **Year** | **1** | **2** | **3** | **4** | **5** | **M** | **25** | **75** |
| --- | --- | --- | --- | --- | --- | --- | --- | --- | --- |
| Being certified by fellow students felt less valuable than being certified by a teacher | 2021 | 8 | 7 | 15 | 9 | 2 | 3 | 2 | 4 |
| Being certified by fellow students felt less valuable than being certified by a teacher | 2022 | 14 | 19 | 17 | 16 | 4 | 3 | 2 | 4 |
| I feel more confident using a light microscope after the certification process | 2021 | 3 | 1 | 4 | 14 | 29 | 5 | 4 | 5 |
| I feel more confident using a light microscope after the certification process | 2022 | 0 | 1 | 7 | 30 | 32 | 4 | 4 | 5 |
| I found the certification unpleasant | 2021 | 20 | 14 | 10 | 5 | 2 | 2 | 1 | 3 |
| I found the certification unpleasant | 2022 | 23 | 16 | 21 | 7 | 3 | 2 | 1 | 3 |
| I think I would have learned just as well with a traditional lab exercise without certification | 2021 | 6 | 20 | 16 | 5 | 4 | 2 | 2 | 3 |
| I think I would have learned just as well with a traditional lab exercise without certification | 2022 | 7 | 27 | 21 | 12 | 3 | 3 | 2 | 3 |
| The certification process increased my motivation to learn how to use a light microscope | 2021 | 2 | 6 | 14 | 17 | 12 | 4 | 3 | 4 |
| The certification process increased my motivation to learn how to use a light microscope | 2022 | 2 | 5 | 20 | 28 | 15 | 4 | 3 | 4 |
| The certification process made me more engaged during the lab exercise | 2021 | 2 | 3 | 9 | 22 | 15 | 4 | 3 | 5 |
| The certification process made me more engaged during the lab exercise | 2022 | 1 | 5 | 17 | 29 | 18 | 4 | 3 | 5 |
| I didn't benefit much from watching the instructional videos before the lab exercise | 2022 | 28 | 30 | 8 | 3 | 1 | 2 | 1 | 2 |
| I felt that I was well prepared for the exercise | 2022 | 0 | 2 | 9 | 38 | 21 | 4 | 4 | 5 |
| I learned nothing from the quiz | 2022 | 35 | 25 | 6 | 3 | 1 | 1.5 | 1 | 2 |
| I think I'm going to watch the instructional videos again if we're going to do microscopy exercises in other topics | 2022 | 5 | 10 | 16 | 28 | 11 | 4 | 3 | 4 |
| I would have learned just as much if the quiz was voluntary | 2022 | 11 | 38 | 10 | 7 | 4 | 2 | 2 | 3 |
| The certification process made me prepare better than usual | 2021 | 4 | 6 | 13 | 16 | 12 | 4 | 3 | 4 |
| The information in the compendium and instructional videos was sufficient to be able to answer the quiz | 2022 | 0 | 6 | 16 | 29 | 19 | 4 | 3 | 5 |
| The information in the instructional videos was more informative than the compendium | 2022 | 1 | 10 | 34 | 16 | 9 | 3 | 3 | 4 |
| The instructional videos increased my understanding of the practical work in the lab | 2022 | 0 | 3 | 9 | 35 | 23 | 4 | 4 | 5 |
| The quiz was a direct reason why I prepared better than usual | 2022 | 2 | 9 | 13 | 29 | 17 | 4 | 3 | 4.25 |
| Certifying others was a waste of time | 2021 | 19 | 14 | 4 | 2 | 2 | 2 | 1 | 2 |
| Certifying others was a waste of time | 2022 | 29 | 16 | 8 | 3 | 0 | 1 | 1 | 2 |
| I learned something myself from certifying others | 2021 | 3 | 1 | 9 | 20 | 8 | 4 | 3 | 4 |
| I learned something myself from certifying others | 2022 | 3 | 4 | 9 | 29 | 11 | 4 | 3 | 4 |
| Certification is a good way to learn practical skills | 2022 | 1 | 2 | 17 | 36 | 14 | 4 | 3 | 4 |
| Certification is an effective way to assess practical skills | 2021 | 2 | 2 | 8 | 25 | 13 | 4 | 3.75 | 5 |
| Certification is an effective way to assess practical skills | 2022 | 0 | 4 | 19 | 37 | 10 | 4 | 3 | 4 |
| Certification is unsuitable for assessing practical skills | 2021 | 18 | 19 | 10 | 2 | 2 | 2 | 1 | 3 |
| Certification is unsuitable for assessing practical skills | 2022 | 16 | 35 | 17 | 2 | 0 | 2 | 2 | 3 |
| Certification should have been used to assess other practical skills as well | 2021 | 6 | 13 | 17 | 8 | 7 | 3 | 2 | 4 |
| Certification should have been used to assess other practical skills as well | 2022 | 3 | 11 | 26 | 24 | 6 | 3 | 3 | 4 |
