# Supplementary material for: “Show me how to use a microscope” – The development and evaluation of certification as direct assessment of practical lab skills
Source: Ecol Evol. 2023 Oct 11;13(10):e10592. doi: 10.1002/ece3.10592 (PMC10568202; doi:10.1002/ece3.10592)
Supplement: Supplementary file 2 — Figure S1 [file ECE3-13-e10592-s002.pdf]

Supplement to: Show me how to use a microscope: development and evaluation of certification as direct assessment of practical lab skills

Pernille Bronken Eidesen[1,2], Anne E. Bjune[3], Simone I. Lang[2]
University of Oslo[1], University Centre in Svalbard[2], University of Bergen[3]
Correspondence: pernilb@uio.no
PO. Box 1066 Blindern, 0316 Oslo, Norway

Supplementary figure 1 Survey responses collected after two iterations (51 in 2021 and 70 in 2022) of practical introduction to light microscopy using a certification assessment at the University of Oslo. The diverging stacked bar chart show the percentage of each response category to statements from 11 questions included in both surveys, separated by year. Number of respondents given to the right. Although the procedure was somewhat adjusted between years, there were no significant differences in how the students responded between years (Appendix 2).

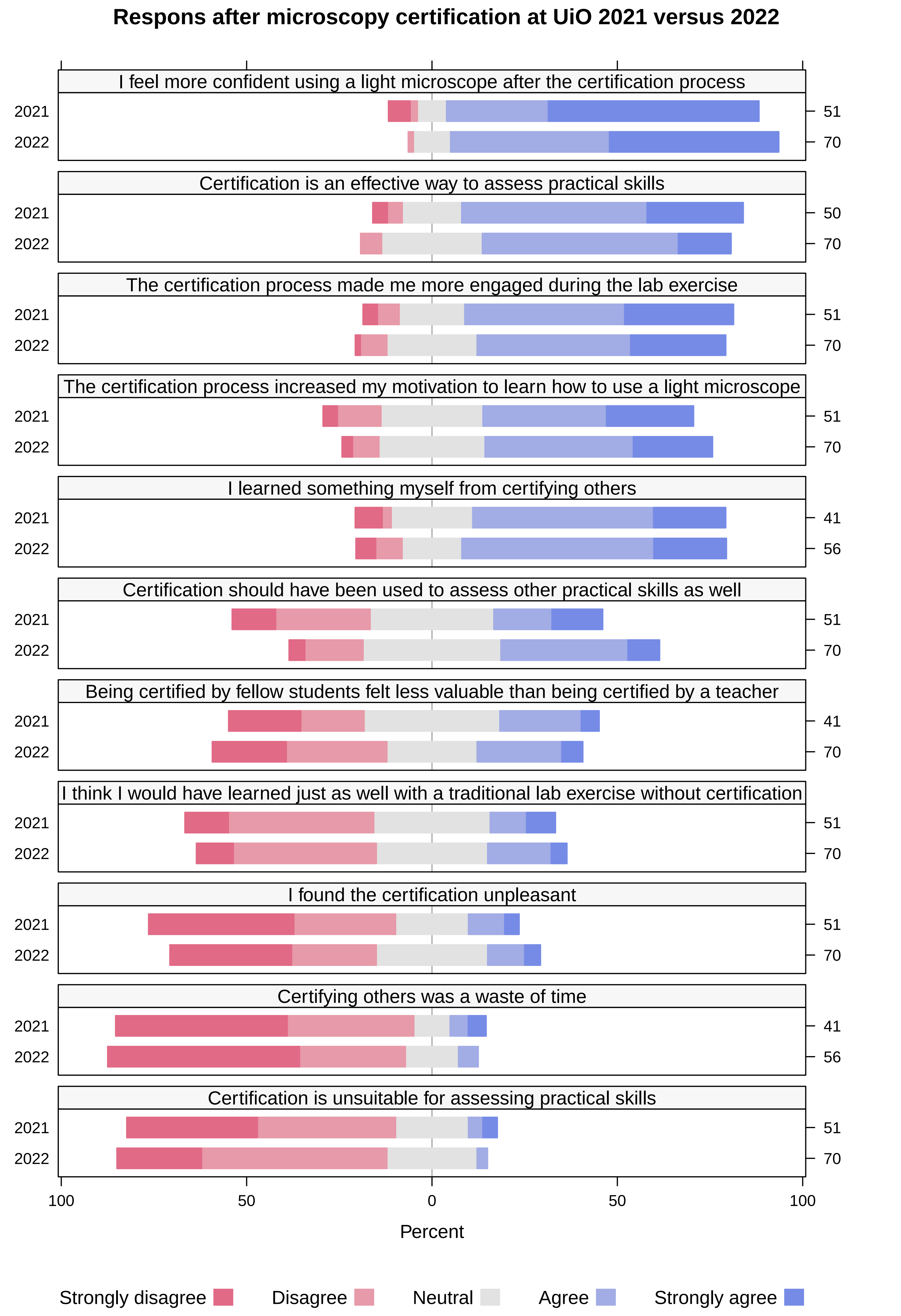
Respons after microscopy certification at UiO 2021 versus 2022
I feel more confident using a light microscope after the certification process
Certification is an effective way to assess practical skills
The certification process made me more engaged during the lab exercise
The certification process increased my motivation to learn how to use a light microscope
I learned something myself from certifying others
Certification should have been used to assess other practical skills as well
Being certified by fellow students felt less valuable than being certified by a teacher
I think I would have learned just as well with a traditional lab exercise without certification
I found the certification unpleasant
Certifying others was a waste of time
Certification is unsuitable for assessing practical skills
2021
2022
51
70
50
70
51
70
51
70
41
56
51
70
41
70
51
70
51
70
41
56
51
70
100
50
0
50
100
Percent
Strongly disagree
Disagree
Neutral
Agree
Strongly agree
